# Electrostatic Comb-Drive Actuator with High In-Plane Translational Velocity

**DOI:** 10.3390/mi7100188

**Published:** 2016-10-17

**Authors:** Yomna M. Eltagoury, Mostafa Soliman, Yasser M. Sabry, Mohammed J. Alotaibi, Diaa Khalil

**Affiliations:** 1 Faculty of Engineering, Ain-Shams University, 1 Elsarayat St. Abbassia, Cairo 11566, Egypt; 2 Si-Ware Systems, 3 Khaled Ibn Al Walid, Qism El-Nozha, Cairo Governorate 11361, Egypt; diaa.khalil@si-ware.com; 3 Department of Power Electronics, Electronics Research Institute, Dokki, Giza 12611, Egypt; mostafa.soliman@eri.sci.eg; 4 National Center of nanotechnology research, King Abdulaziz City for Science and Technology, Riyadh 11442, Saudi Arabia; mjalotaibi@kacst.edu.sa

**Keywords:** microelectromechanical system (MEMS) actuator, deep reactive ion etching (DRIE), optical MEMS, high speed tunable filter, optical fringes

## Abstract

This work reports the design and opto-mechanical characterization of high velocity comb-drive actuators producing in-plane motion and fabricated using the technology of deep reactive ion etching (DRIE) of silicon-on-insulator (SOI) substrate. The actuators drive vertical mirrors acting on optical beams propagating in-plane with respect to the substrate. The actuator-mirror device is a fabrication on an SOI wafer with 80 μm etching depth, surface roughness of about 15 nm peak to valley and etching verticality that is better than 0.1 degree. The travel range of the actuators is extracted using an optical method based on optical cavity response and accounting for the diffraction effect. One design achieves a travel range of approximately 9.1 µm at a resonance frequency of approximately 26.1 kHz, while the second design achieves about 2 µm at 93.5 kHz. The two specific designs reported achieve peak velocities of about 1.48 and 1.18 m/s, respectively, which is the highest product of the travel range and frequency for an in-plane microelectromechanical system (MEMS) motion under atmospheric pressure, to the best of the authors’ knowledge. The first design possesses high spring linearity over its travel range with about 350 ppm change in the resonance frequency, while the second design achieves higher resonance frequency on the expense of linearity. The theoretical predications and the experimental results show good agreement.

## 1. Introduction

Creating micro-optical benches using the microelectromechanical system (MEMS) technology is one of the MEMS promises that can lead to a critical optical microsystems development. In this direction, different in-plane optical MEMS components have been proposed and tested recently using the deep reactive ion etching (DRIE) Technology [1]. This direction allows building a complete optical system on a substrate with all the components self-aligned in the in-plane direction with the lithography resolution. In addition, the actuation of these components using high speed MEMS actuators paves the road for new applications of significant academic and industrial impact such as, integrated wide angle optical microscanner [2], high-speed swept laser sources for optical cohere tomography application [3], hand-held Fourier transform infrared spectrometers [4] among others. For instance, increasing both frequency and travel range of the MEMS actuators leads to high frame rate for the scanner while achieving wide scanning angle, high wavelength sweeping speed for the laser source while achieving the tunability range needed for deep medical imaging applications and, finally small acquisition time for the spectrum with high resolution in case of the optical spectrometers. Therefore, there is a need for MEMS actuators with a long travel range and high frequency, simultaneously.

The resonance frequency of the MEMS actuator is typically represented by the fundamental mechanical mode frequency, after which the force-to-displacement transfer function shows significant attenuation. However, the increase of the resonance frequency is usually associated with the decrease of the actuator travel range due to the high spring stiffness and short beams lengths leading to less linearity and the need of larger applied force. Therefore, the authors suggest the use of the product of the resonance frequency and the amplitude of the travel range; i.e., the maximum stroke, as a “Figure of Merit” (FOM) for the actuator performance with units of µm·kHz. This FOM is proportional to the peak translation velocity, which is given by $\nu_{\mathrm{peak}}=2\pi f_{r}S$ where $f_{r}$ is the resonance frequency and *S* is the maximum stroke.

Both Thermal and magnetic actuators can usually achieve long travel ranges but with very limited speeds [5,6]. On the other hand, piezoelectric actuators are used for short travel range with small resolution [7]. Focusing on electrostatic actuators, we can identify mainly two structures: the comb-drive actuator and the parallel plate actuator. The first is usually used for long travel range applications with relatively slow response while the second is usually characterized by a high speed response with very limited travel range [8] due to its front instability. In addition, we consider the operation under atmospheric pressure since we target applications with in-plane integrated MEMS and optical fiber components, where the latter are inserted into micromachined groves with open access from the outside of the chip [9]. Similar conditions are needed for optofluidic chips, where additional micro tubes for fluid handling are used [10]. This condition puts challenging limitation due to air friction that limits the quality factor of the MEMS resonator and hence its maximum travel range. Within these operating conditions, the maximum achieved FOM is about 138.5 µm·kHz reported by John D. Grade et al. [11]. A comprehensive survey on the electrostatic actuators is out of scope of this work but a quick survey is given in Table 1, in descending order according to the FOM, to show the tradeoff between the resonance frequency and the travel range. It is noteworthy that out-of-plane MEMS actuator can achieve larger FOM as reported in [12,13,14,15]. This is due to the smaller etching depth leading to more compliant springs and overall structure with less weight but preventing the monolithic integration of different optical components on-chip. 

In this paper, we report two in-plane MEMS comb-drive actuators working under atmospheric pressure with FOMs of about 236 and 187 µm·kHz. The corresponding peak velocities are about 1.48 m/s and 1.18 m/s, respectively. The FOM depends on both the design’s physical parameters and the applied voltage. The quality factor is increased by increasing the actuator’s natural frequency, but not to very high values in order to avoid thermal-structural energy dissipation in the beams due to bending at high frequencies. In addition, the spring is designed to achieve high linearity to reduce the energy dissipation due to non-linearity. However, as will be discussed later, the FOM appears to be proportional to the physical size of the structure and inversely proportional to the fluid viscosity, increasing the size of the MEMS structure leads directly to larger area, higher damping coefficient and, thus smaller FOM. Therefore, the size of the device is optimized to achieve high FOM. The release holes in the structures are designed to decrease area while assisting the release process and, thus increase FOM. Decreasing the gap has the overall effect of increasing the FOM, although the damping coefficient increases.

The rest of this article is organized as follows. Section 2 discusses the design constraints along with the design approach, considerations, and selected actuator geometry. In Section 3, the design of the actuators and the finite element (FE) simulation results are presented. The fabrication steps and the resulting structures are given in Section 4. The opto-mechanical characterization method is introduced and explained in Section 5. Finally, the experimental results are reported and discussed in Section 6.

## 2. Actuator Design

The objective of the design is to increase the product of the MEMS actuator maximum travel range and its resonance frequency. The design constraints can be summarized as follows:

(1) Atmospheric pressure for compatibility with the micro-optical benches and lab-on-chip microsystems.
(2) Actuation with direct current (DC) voltage of 100–150 V and alternating current (AC) voltage less than 10 V.
(3) Maximum stress in flexure beams is less than 1% of the material’s Young’s modulus to avoid fracture [27].
(4) Compatibility with optical MEMS technology with in-plane optical axis, for instance with DRIE of silicon-on-insulator (SOI) wafers.

Scanning the different types of high speed electrostatic MEMS actuators, one can identify two main categories, the comb-drive actuator and the parallel plat actuator [8]. Although parallel plate actuators can operate at very high frequencies with maximum travel stroke of 1 µm or less, such actuators suffer from a very high squeeze film damping of the air between the moving and the fixed plates that reduces the quality factor under atmospheric pressure; and thus the maximum travel range will be limited. The parallel plate actuator suffers as well from front instability due to the pull-in phenomenon [28]. The authors have reported previously a novel design with perforated fixed plate that could achieve a travel range of 0.8 µm and a resonance frequency of 90 kHz in a previous work [29]. In contrast, comb-drive actuators are chosen to achieve the target of the current work, as they can operate with frequencies higher than 10 kHz and with a travel range that is longer than 100 µm. Comb-drive actuators are characterized also by [8] good linearity over the travel range in the sense that the forces is independent on the overlap length between the fingers once the combs are engaged, high electro-mechanical stability, and relatively small damping coefficient limited by slide film damping rather than squeeze film damping. Such characteristics of the comb-drive actuators make them favorable designs for our target of high FOM and relatively long travel range MEMS actuator.

**Figure 1:**
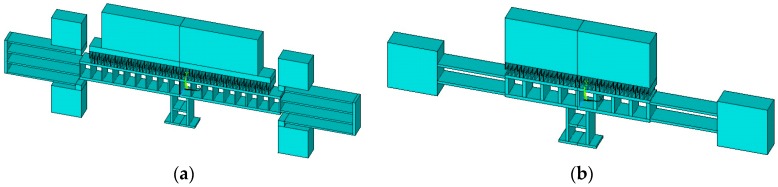
Schematic three-dimensional representation of the comb-drive actuators: (**a**) Design A using folded beam spring and (**b**) Design B using fixed-fixed spring.

In the following, the dependence of the FOM on the design parameters is discussed followed by the selection of the spring and the linearity dependence. Taking these points into considerations, two designs have been developed for the actuator as shown in Figure 1. The details of the two designs as well as their finite element analysis (FEA) performed using ANSYS software (version 14, ANSYS Inc., PA, USA) will be given in Section 3. Design A makes use of a folded-beam spring to maintain the linearity of the design achieving large stroke, while design B utilizes the more compact fixed-fixed spring but shows larger nonlinearity.

### Figure of Merit

The actuators are designed to operate at resonance frequency. Therefore, the stroke or the full travel range (FTR) will be multiplied by the quality factor; i.e., x=QF/k where *k* is the spring stiffness, *F* is force applied on actuator, the quality factor $Q=\sqrt{Km}/b$, where *m* is the effective oscillating mass and *b* is air damping coefficient [30], which is approximately proportional to the mass of the actuator. The resonance frequency is given by $f_{r}=\sqrt{k/m}/2\pi$ and, thus the FOM is given by:

(1) $$\mathrm{FOM}=\mathrm{FTR}\times f_{r}=\frac{F_{r}}{2\pi b}$$

where *F*_r_ is force applied at resonance frequency. The FOM is controlled by the force and damping coefficient, which includes the geometrical parameters. A combination of DC and AC voltage (*V*_ac_ + *V*_dc_) is applied on the comb-drive and the resulting electrostatic force will act on the moving fingers set, which is suspended by the anchored spring. The electrostatic force is given by [31]:

(2) $$F=\frac{n\varepsilon t_{s}}{g}\left({V_{\mathrm{dc}}}^{2}+\frac{{V_{\mathrm{ac}}}^{2}}{2}\right)+\frac{n\varepsilon t_{s}}{g}\left(\frac{{V_{\mathrm{ac}}}^{2}}{2}\cos\left(2\omega_{r}t\right)\right)+\frac{n\varepsilon t_{s}}{g}\left(2V_{\mathrm{dc}}V_{\mathrm{ac}}\cos\left(\omega_{r}t\right)\right)$$

where *n* is the number of moving fingers, ε is the permittivity of free space, *t*_s_ is the thickness of silicon or the etching depth, *g* is the gap between the fixed and moving finger, and ω_r_ is the resonant angular frequency of the actuator. The first term is a DC term leading to static motion, the second term is excitation at twice the resonance frequency and the third term is the excitation at the resonance frequency. The DC component and the component at twice the excitation frequency will lead to negligible displacement compared to the resonance term amplified by the quality factor. The force at resonance is then given by:

(3) $$F_{r}=\frac{n\varepsilon t_{s}}{g}\left(2V_{\mathrm{dc}}V_{\mathrm{ac}}\cos\left(\omega_{r}t\right)\right)$$

The total air damping coefficient for comb-drive actuators can be expressed as [28]:

(4) $$b={µ}_{\mathrm{air}}\left[\frac{A_{\mathrm{eff}}}{d_{{\mathrm{sio}}_{2}}}+\frac{A_{\mathrm{eff}}}{\delta }+\frac{A_{s}}{g}+10.7L\right]$$

where μ_air_ is the viscosity of the air, *A*_eff_ the effective plate area of design, including the areas of the plates, fingers and beams, *A*_s_ is the side area of actuator, $d_{{\mathrm{sio}}_{2}}$ is the thickness of silicon dioxide underneath the moving structure, *L* is the characteristic dimension of the moving structure that maybe be taken as half the width of the plate, and δ is the effective decay distance given by:

(5) $$\delta =\sqrt{\frac{2{µ}_{\mathrm{air}}}{\rho_{\mathrm{air}}\omega }}$$

where ρ_air_ is the density of air. The actuator’s FOM depends on both the physical parameters and the applied voltage of the design. The best approach to obtaining structures with high quality factors is by increasing the natural frequency by increasing the spring stiffness and decreasing the mass as much as possible. However, the operation at very high resonant frequencies may depreciate the quality factor, as another energy dissipation factor appears which reduces the quality factor. This energy dissipation factor is the thermal-structural dissipation in the beams due to bending at high frequencies which leads to the importance of the spring linearity [30] that will be discussed hereinafter. It appears that the FOM is proportional to the physical size of the structure (number of fingers and thickness) and inversely proportional to the fluid viscosity (air in this case). However, increasing the size of the MEMS structure leads to larger area, higher damping coefficient and, thus, smaller FOM. Therefore, increasing the number of fingers has weak impact on the FOM. The release holes in the structures are designed to decrease area while assisting the release process and, thus, increase FOM. Decreasing the gap has the overall effect of increasing the FOM, although the damping coefficient increases.

In order to maximize the travel range of the MEMS actuator, it is required to operate the MEMS actuator at resonance. This means the actuator’s natural frequency should be fixed over the whole travel range of the actuator. However, due to spring nonlinearity, spring stiffness may change with the travel stroke, softening occurs when spring stiffness decreases with displacement while spring stiffness increases with displacement due to hardening effect; and this in return will limit the expected travel stroke of the actuator [30,32]. Thus, the resonance frequency of MEMS actuator will increase with increasing travel stroke. The spring force-displacement relation can be written as:

(6) $$F=kx+k_{1}x^{2}+k_{2}x^{3}+O\left(x^{4}\right)$$

where *k* is the linear spring constant, *k*_1_ and *k*_2_ are the first and second order corrections, respectively, *x* is the spring displacement, and $O\left(x^{4}\right)$ is function in $x^{4}$ and higher orders. The shift in oscillation frequency depends on the values of *k*_1_ and *k*_2_; for positive values of *k*_1_ and *k*_2_, which means effective spring stiffness increases, so the resonance curve is bent towards larger frequencies, while for negative values, the bent is toward the smaller frequencies. This effect will be studies carefully in design B, which exhibits non-linearity expected theoretically and measured experimentally.

## 3. Finite Element Analysis

### 3.1. Design A

Table 2 lists all the physical dimensions of this design. In order to simplify the FEA, the force has been distributed on the tips of the moving fingers without the need to consider the fixed finger set in the analysis; to reduce the number of elements/nodes required for the simulations. The springs are designed to be stiff with small oscillating mass and with wide release holes. The release holes are not too wide to avoid deformation in the moving mass frame over the whole travel range of the actuator and to reduce the possibility of comb fingers side instability.

**Figure 2:**
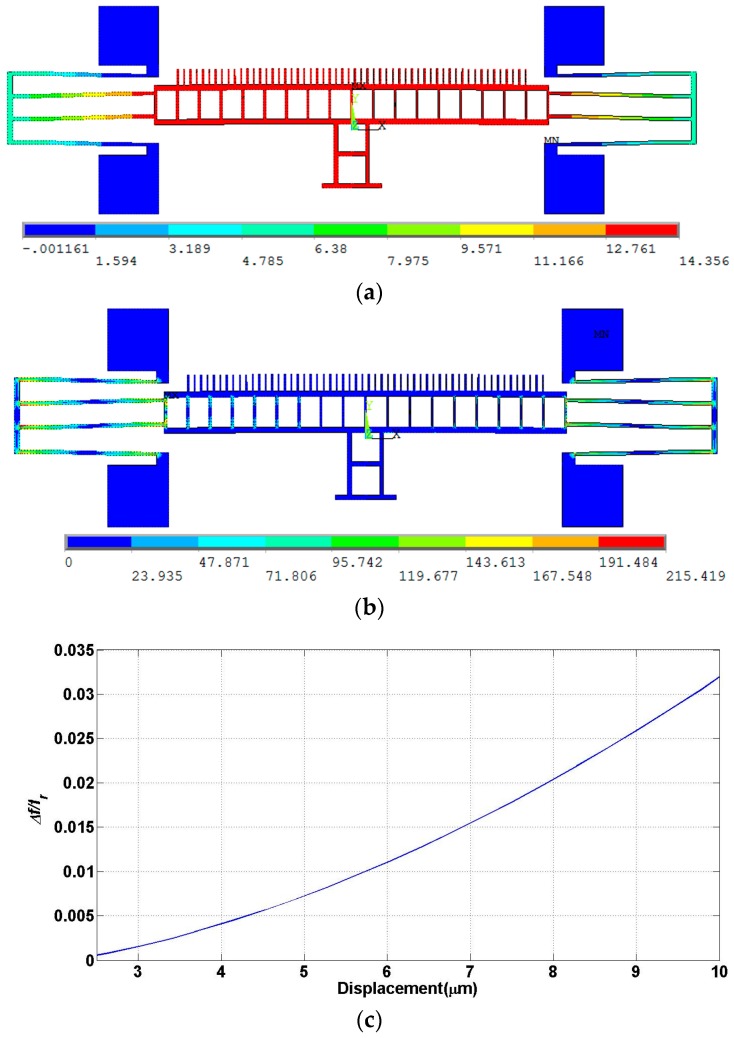
Finite element analysis (FEA) of design A: (**a**) Actuator displacement in µm; (**b**) Stress profile in µN/µm^2^ and (**c**) Resonant frequency shift percentage, percentage of difference in frequency divided by resonance frequency *f*_r_, versus displacement.

Figure 2a shows the static nonlinear FE analysis of the actuator design. It shows the actuator displacement of the moving mass for a stroke of 14 µm in the motion direction perpendicular to the mirror surface. There is no deformation in the moving mass and the maximum stress (~215 µN/µm^2^) in the beams does not exceed the maximum allowed fracture stress (1690 µN/µm^2^) as shown in Figure 2b. Figure 2c depicts the shift in the resonant frequency of the actuator when its travel stroke increases from 2 to 19 µm due to the hardening effect. This is deduced by finding the stiffness of the spring, and hence the corresponding resonance frequency, using both linear and nonlinear FE analyses. The shift in the resonant frequency in the desired operating range (2.5~10 µm) is only 10 Hz, normalized difference less than 3.5 × 10^−4^ (0.035%), which indicates very good linearity of the suspension spring system.

**Figure 3:**
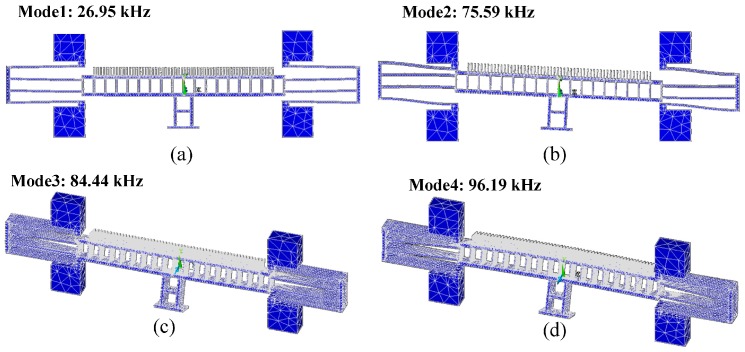
The first four modes of design A: (**a**) The first mode in-plane at 26.95 kHz; (**b**) The second mode in-plane at 75.59 kHz; (**c**) the third mode out-of-plane at 84.44 kHz; while (**d**) The fourth mode out-of-plane at 96.19 kHz.

Modal analysis was performed to extract the modes of the structures and their Eigen values. Figure 3a,b depicts the first and the second modes at frequencies of 26.95 and 75.59 kHz, respectively. These are in-plane modes while the third and the fourth modes shown in Figure 3c,d at frequencies 84.44 and 96.19 kHz, respectively are out of plane modes. The frequency of the second mode is far enough from the second harmonic of the excitation frequency at the first mode resonance. This is important to consider as the force applied on the actuator will excite the fundamental frequency and its second harmonic as explained by Equation (3).

### 3.2. Design B

**Figure 4:**
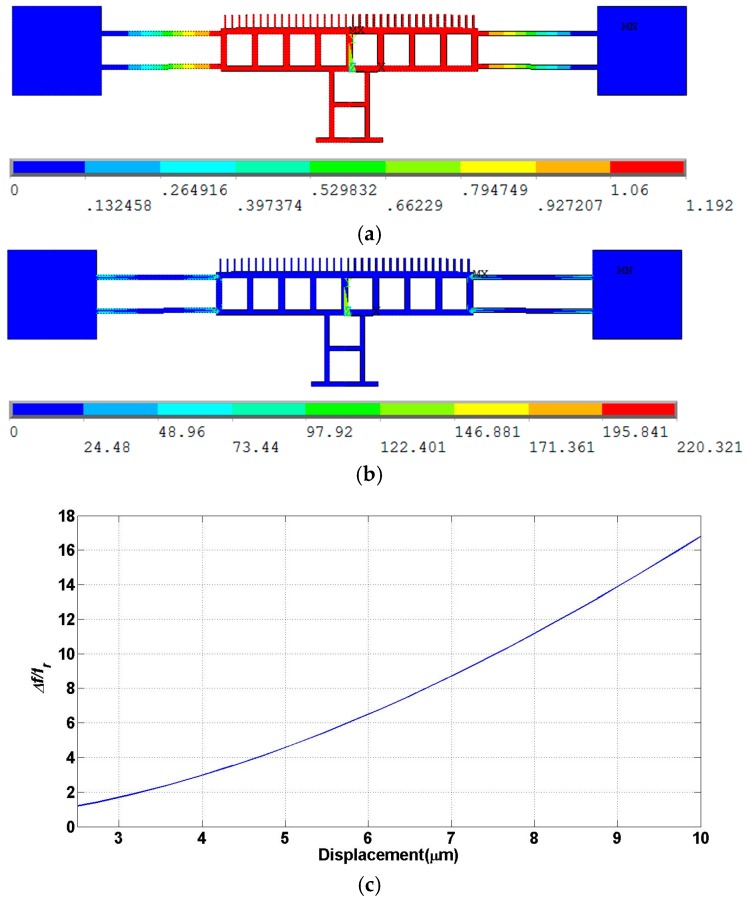
FEA of design B: (**a**) Actuator displacement in µm; (**b**) Stress profile in µN/µm^2^ and (**c**) Resonant frequency shift percentage, percentage of difference in frequency divided by resonance frequency *f*_r_, versus displacement.

**Figure 5:**
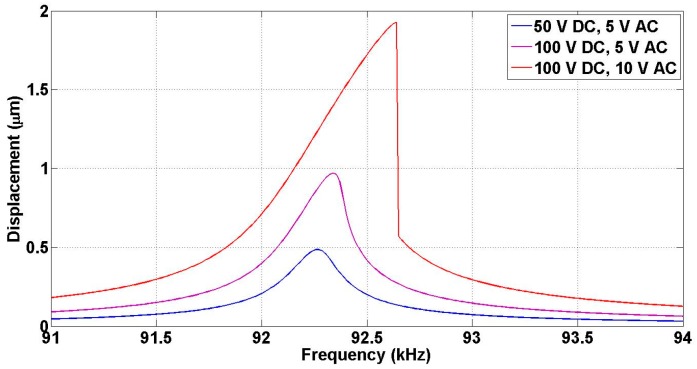
Frequency response of the non-linear MEMS actuator system for different applied voltages.

**Figure 6:**
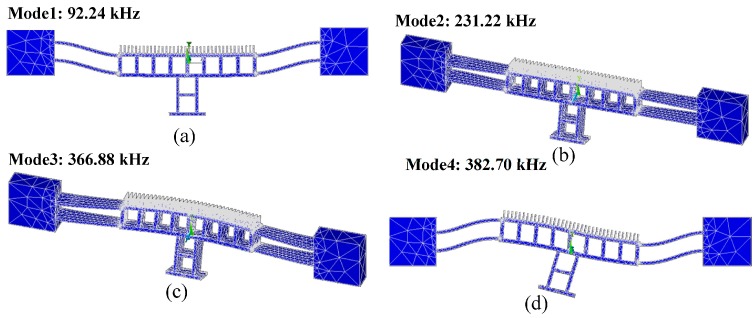
The first four modes of design B: (**a**) The first mode in-plane at 92.24 kHz; (**b**) The second mode out-of-plane at 231.22 kHz; (**c**) the third mode out-of-plane at 366.88 kHz; while (**d**) The fourth mode in-plane at 382.70 kHz.

The physical dimensions of this design are listed in Table 3. Fixed-fixed suspension beams were used due their high stiffness. Again, the size of the release holes is chosen such that the design is rigid along the whole travel range. Similar to design A, FEA was carried out on design B as shown in Figure 4. The static nonlinear FEA shows the actuator displacement of the moving mass for a stroke of about 1.2 µm. The design is rigid enough and no deformation is observed in the moving mass. The maximum stress (~220 µN/µm^2^) in the beams is safe and below the fracture limit. Figure 4c shows the frequency shift of the resonance frequency due to the hardening effect of the spring. The normalized frequency difference is less than 0.017 (1.7%) at 3 µm travel range, in which the non-linearity effect is significant. It is important to get the frequency response of the displacement by solving second order system differential equation of motion and accounting for the non-linearity of the spring [32]. The nonlinear terms of spring constants $k_{1}$ and $k_{2}$ can be deduced by fitting the FEA result on Equation (6). The coefficients were obtained to be *k*_1_ = 1.7 × 10^7^ N/m and *k*_2_ = 6.7 × 10^12^ N/m. The frequency response is shown in Figure 5 at different applied voltages and assuming a quality factor of 400. The figure depicts the shift in resonance frequency for 50 V DC/5 V AC, 100 V DC/5 V AC and 100 V DC/10 V AC. Finally, modal analysis was performed and the resonance frequencies were calculated as shown in Figure 6. The first and fourth modes are in-plane with resonance frequencies of 92.24 and 231.22 kHz, respectively, while the second and third modes are out-of-plane with resonance frequencies of 382.70 and 366.88 kHz, respectively.

## 4. Device Fabrication

**Figure 7:**
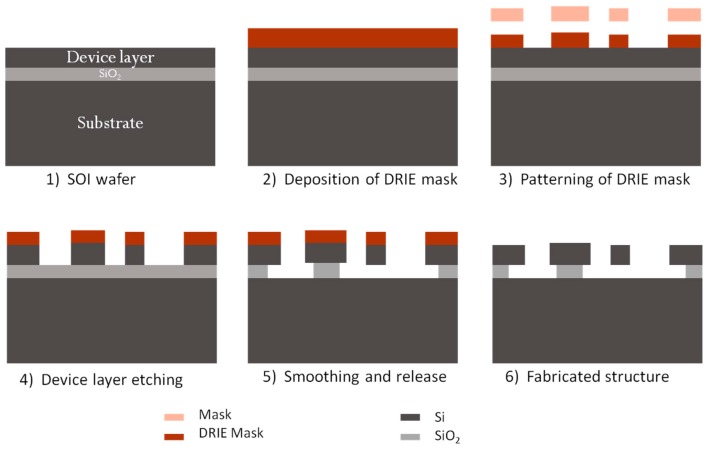
The main fabrication steps of the MEMS spectrometer.

**Figure 8:**
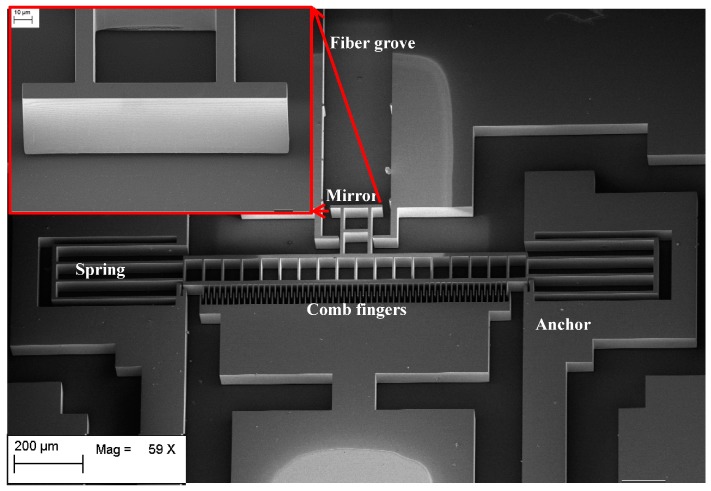
An scanning electron microscope (SEM) image of design A MEMS actuator with the insert showing a close-up of the micromirror surface prior to gold metallization. (Scale bar 10 μm (**inset**))

**Figure 9:**
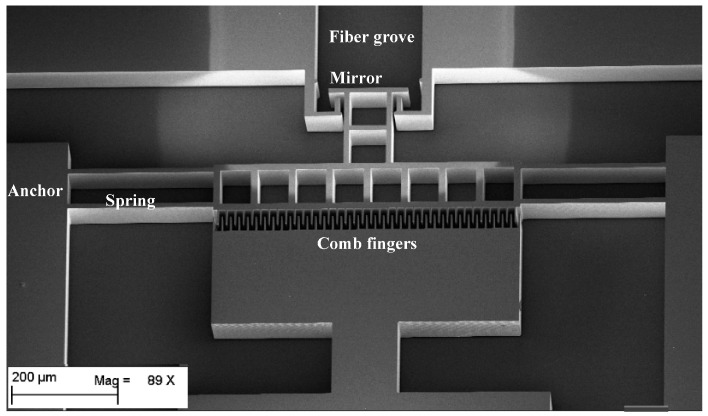
An SEM image of design B MEMS actuator.

The MEMS devices were fabricated using deep reactive ion etching (DRIE) technology on an SOI wafer in a monolithically integrated manner. The device layer height of the SOI is 80 µm and the buried oxide (BOX) layer thickness is 3 µm. Figure 7 shows the main fabrication steps. The movable parts are released by etching the oxide layer underneath the structures using vapor HF etching of the SiO_2_. Fiber groove are used to guide the fiber inside the optical MEMS chip in a self-aligned manner. The MEMS actuators are driving a micromirror that is used for the opto-mechanical characterization of the devices making use of an optical cavity between the mirror and the optical fiber surfaces. The scanning electron microscope images of the fabricated structures are shown in Figure 8 and Figure 9. To minimize the surface roughness, post-etching smoothing of the optical surfaces is applied to produce a micromirror with high surface quality. The optical surface was obtained with a sidewall angle whose deviation from the perfect verticality is smaller than 0.1°. The peak-to-peak surface roughness of the micromirror is in the order of 60 nm. The micromirror surface is shown in the insert of Figure 8.

## 5. Opto-Mechanical Characterization Method

**Figure 10:**
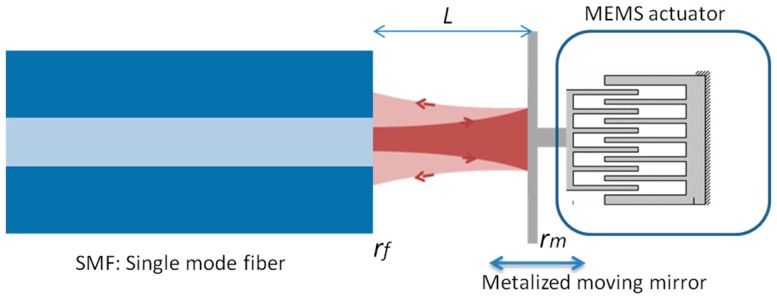
Schematic representation of the fiber Fabry-Perot interferometer with metalized moving mirror connected to MEMS actuator.

There are many out-of-plane measurement techniques, such as laser Doppler velocimetry [33], stroboscopic imaging [34] and laser feedback interferometry [35,36] but an in-plane measuring technique was preferred for compatibility with the target micro-optical bench technology. An optical test method to characterize the mechanical displacement of the high speed MEMS actuators was developed [37]. The technique is based on optical interferometry. Basically, if two optical beams with a phase shift between them are combined, they produce an interference signal that holds information about the value of the phase shift between the two beams. In our case, the displacement is used to produce a phase shift. To measure the displacement of the MEMS actuator, a micromirror is attached to the actuator as shown in Figure 8 and Figure 9. Considering a laser beam output from an optical fiber inserted in the micromachined fiber groves, the beam is travelling in-plane with respect to the substrate. The laser beam reflected from the MEMS mirror will have a different phase for each mirror position relative to the beam reflected from the fiber end face. The optical arrangement of the MEMS mirror with reflection coefficient *r*_m_ and the fiber end face with reflection coefficient *r*_f_ serves as a low finesse Fabry-Perot interferometer as shown in Figure 10. By inspecting the resulting interference signal against the mirror movement, information about the displacement of the actuator can be extracted as will be explained hereinafter [38].

### 5.1. Analysis of the Characterization Method

Assuming the single-mode fiber (SMF) output is a Gaussian beam, which travels through the air cavity and reflected back, the diffraction effect cannot be neglected. The overall reflection coefficient of a fiber Fabry-Perot cavity with two interfaces having reflection coefficients *r*_f_ and *r*_m_ and a cavity length L accounting for the Gaussian beam diffraction is given by the summation [39]:

(7) $$r_{\mathrm{FP}}=r_{f}+\frac{1-{\left|r_{f}\right|}^{2}}{r_{f}}\sum_{m=1}^{\infty }\frac{r_{m}^{m}r_{f}^{m}}{1-jmL/z_{o}}\exp\left[-j\frac{2\pi }{\lambda }2mL\right]$$

where *m* is the round trip number, λ is the wavelength of the light and *z_o_* is the Gaussian beam’s Rayleigh range. Separating the reflected signal by means of a directional coupler, the optical detector current is given by:

(8) $$I_{\mathrm{out}}\sim {\left|r_{\mathrm{FP}}\right|}^{2}$$

**Figure 11:**
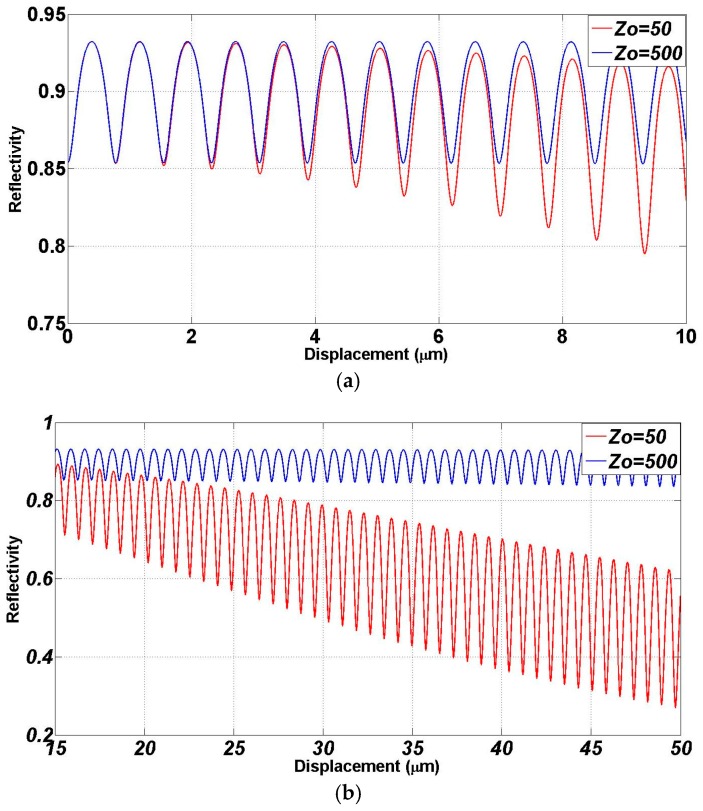
Simulated result of obtained interferogram suing the low-finesse Fabry-Perot using Gaussian beam with Rayleigh range *z_o_* of 50 and 500 µm: (**a**) 10 µm displacement of the micromirror in one direction; and (**b**) from 15 to 50 µm motion.

The variation of the current versus cavity length given by Equation (8) represents the output interferogram. As the reflection from the fiber end is quite weak due to the small difference in the refractive index between the silica and the air, the Fabry-Perot interferometer is approximately acting as a two beam interferometer (or a Michelson interferometer) and the resultant fringes are expected to be nearly sinusoidal with a periodicity of half the wavelength. By counting the peaks (number of maxima’s) of the interference signal and multiplying by half the wavelength one can get MEMS traveled distance (*x*). Equation (8) is plotted for *R*_f_ = *r*_f_^2^ = 4% (silica-air interface) and metalized mirror with reflection coefficient intensity *R*_m_ = *r*_m_^2^ = 90%, and for different Rayleigh ranges of 50 and 500 µm to analyze the diffraction effect. The case of 50 μm represents the practical case of using the standard SMF 28. The results are shown in Figure 11 and show that the larger the Rayleigh range, the larger tendency to the plane wave incidence cavity response with no degradation in the mean value of the interferogram versus cavity length. In addition to this degradation for small Rayleigh range shown at larger distances in Figure 11b, there is small shift in the peak locations as shown in Figure 11a. The periodic displacement of the plot is about λ/2.

### 5.2. Characterization Setup 

**Figure 12:**
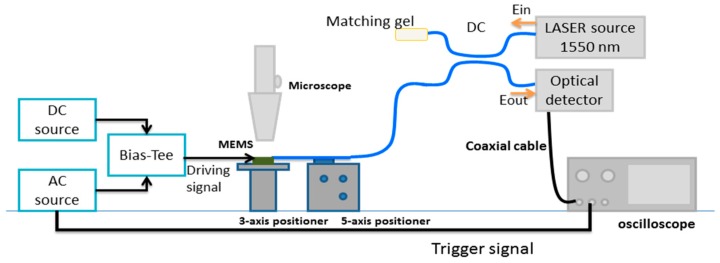
Optical characterization setup of the MEMS actuators using Fabry-Perot interferometer between the optical fiber and the micromirror attached to the MEMS actuators.

The testing setup illustrated schematically in Figure 12 was built on an optical table for vibration isolation. The MEMS sample was placed on a micro-positioner for optical alignment in front of the fiber tip. The input laser with a wavelength of 1550 nm was coupled into the SMF by means of an optical directional coupler. The back reflected signal was separated using the directional coupler and connected to the detector. The electrical driving AC sinusoidal signal of the MEMS actuator was provided from a function generator, and a DC voltage source for providing the DC signal was used. A bias-tee was used to combine the AC and the DC voltage signals. The SMF was fixed on a 5-axis micro-positioner for both linear and angular adjusted to placement in the fiber groves. The non-used port of the coupler was terminated with matching gel to minimize unwanted reflections. An oscilloscope was connected to the detector output to display the interference signal in the electrical domain. The interference signal is nearly a cosine function with a periodicity half the wavelength as described before. By counting the peaks of the interference signal, the stroke of the MEMS actuator could be determined roughly and then use this initial value for fitting of the theoretical equation on the experimental data.

## 6. Experimental Results

### 6.1. Mirror Travel Range

**Figure 13:**
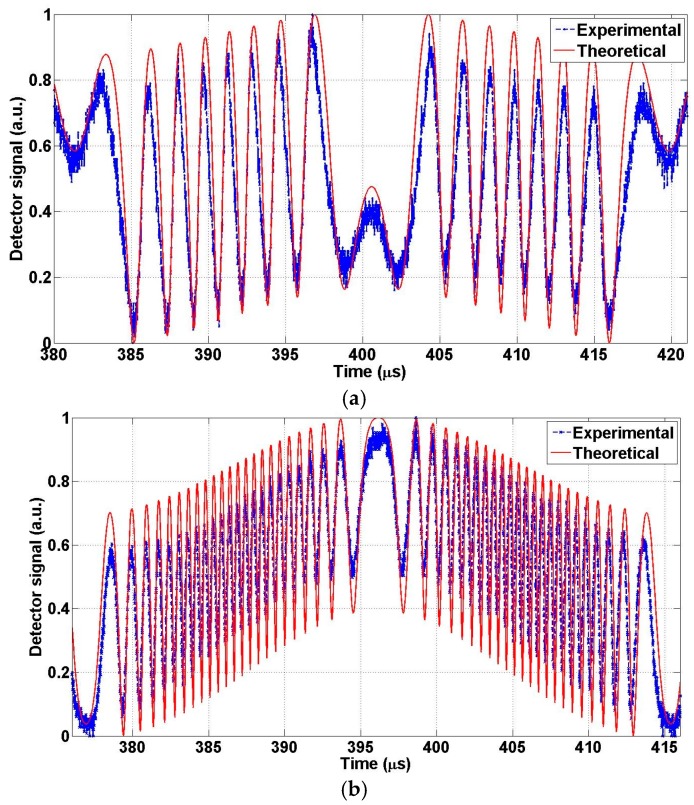
Optical interferogram obtained using actuator A and the corresponding theoretical response using best fit for the actuator travel range. (**a**) Under applied voltage of about 36 V direct current (DC) and 9.5 V alternating current (AC); (**b**) Under applied voltage of about 114 V DC & 4.5 V AC.

The objective of this test is to determine the maximum mechanical FTR of the actuator when operated at a given frequency and finding the maximum product of the FTR and the frequency at resonance. Actuator A was connected using the setup shown in Figure 12. Figure 13a depicts the experimental interferogram recorded due to the motion of the MEMS mirror in front of the fiber. The applied voltage for this test was recorded to be 36 V DC and 9.5 V AC at a fixed frequency of 26.06 kHz. The results are fitted to the theoretical predictions. Figure 13 shows the effect of diffraction. As discussed in Section 5.1, the signal intensity is different for opposite directions around the rest position. The difference in the contrast of the interferogram between theoretical and experimental may be due to misalignment due to the fiber cleaving angle. The peak-to-peak displacement, maxima to maxima or minima to minima, travelled by the actuator can be roughly estimated by fringe counting as given by:

(9) $$\mathrm{Displacement}=\text{Number of peaks}\times \frac{\lambda }{2}$$

For accurate prediction, fitting of the measured data and the theoretical analysis using Equation (7) was used, which also includes the effect of diffraction. The estimated displacement from the fitting is 3.07 µm, which means that the FTR equals to 6.14 µm. Increasing the applied voltage, while fixing the frequency at 26.06 kHz, increases the number of fringes measured by the oscilloscope. Figure 13b shows an optical full travel range is of 18.12 µm when using a voltage of 4.5 V AC and 114 V DC. This optical path difference is equivalent to 9.06 µm mechanical FTR. Therefore, the FOM is given by:

$$\mathrm{FOM}=\mathrm{FTR}\times f_{r}=\frac{18.12}{2}\times 26.06=236\;\mu m\cdot \mathrm{kHz}$$

**Figure 14:**
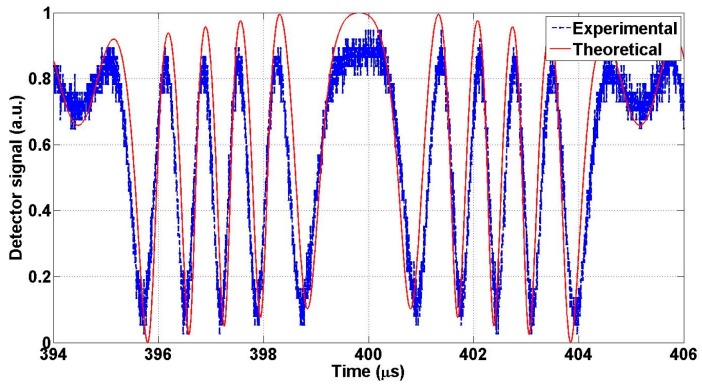
Optical interferogram obtained using actuator B at 150 V DC and 10 V AC and the corresponding theoretical response using best fit for the actuator travel range.

This is largest FOM reported in literature for an in-plane optical MEMS actuator, up the author’s knowledge. The same procedure of this test was repeated on design B, where the results are shown in Figure 14 for applied voltage of 150 V DC and 10 V AC at frequency 93.5 kHz. One can find about 4 µm motion in one direction. Therefore, the FOM of this actuator is given by:

$$\mathrm{FOM}=\mathrm{FTR}\times f_{r}=\frac{4}{2}\times 93.50=187\;\mu m\cdot \mathrm{kHz}$$

This FOM also exceeds the reported values in literature and it has the advantage of being at higher frequency than design A.

### 6.2. Frequency Response

**Figure 15:**
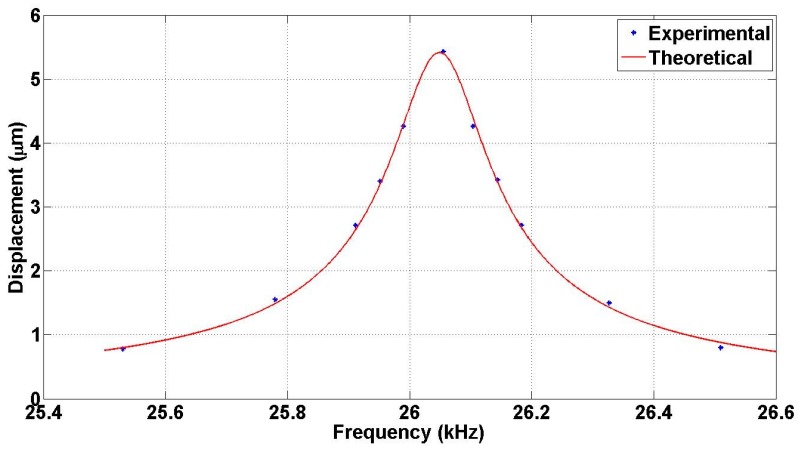
Mirror displacement as a function of the AC signal frequency at applied voltage of 70 V DC and 8.5 V AC for design A.

The main objective of this test is to determine the resonance frequency and the quality factor of the actuator. The resonance frequency is obtained by sweeping the frequency of the applied AC voltage while keeping the amplitude of both the DC and the AC voltages constant. For design A, the DC and AC voltage levels in this test were about 70 and 8.5 V, respectively. The frequency is swept from 25.5 to 26.5 kHz. The resonance frequency was identified from the resulting signal on the oscilloscope as the frequency, at which the number of fringes in the interferogram is maximized and was found to be as 26.06 kHz. This is the value at which the FOM was found in the previous section. The measured resonance frequency is in good agreement with the simulation results that indicates a resonance at 26.95 kHz. This slight difference can be due to in over etching in the deep etching process. The frequency response of the actuator was recorded and compared to the theoretical response of the second order system as shown in Figure 15. The quality factor is found to be 170.

**Figure 16:**
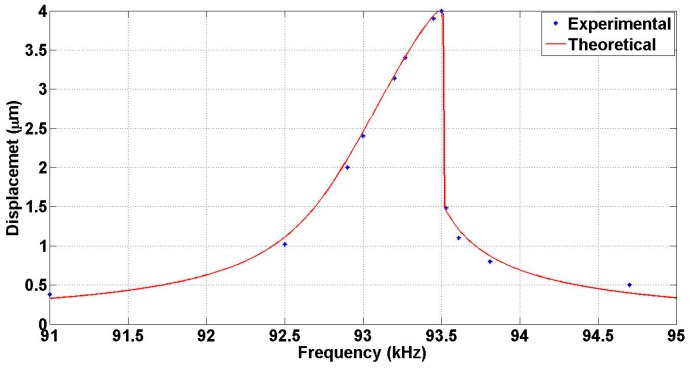
Displacement vs. frequency at 150 V DC &10 V AC for design B Mirror travel range.

The resonance frequency of design B was measured by sweeping the frequency from 91 to 94.6 kHz at DC and AC voltages of 150 and 10 V respectively. It is found to be 93.50 kHz, as shown in Figure 16, in good agreement with the simulation results presented in Section 3. The quality factor is then determined from Figure 16 by fitting the experimental data to the theoretical prediction in the presence of non-linearity and was found to be 282.

## 7. Conclusions

The design, fabrication and characterization of a high-speed MEMS actuator with in-plane motion have been detailed in this article, reporting the highest product of the travel range and frequency for an in-plane MEMS motion under atmospheric pressure. The in-plane actuators are capable of driving micromirrors for micro-optical bench applications, in which the light is propagating in-plane and parallel to the substrate. Two actuators were reported having resonance frequencies of about 26.1 and 93.5 kHz and achieving a single-side travel range of about 9.1 and 2 µm, respectively with different linearity performances. An opto-mechanical characterization method was used accounting for the diffraction effect in the microscale. The method was used to accurately predict the displacement of the micromirrors and to extract the frequency response of the actuators. All the experimental results were compared to the theoretical analysis and possessed good agreement. In contrast to out-of-plane MEMS actuators, the reported devices allow building a complete optical system on a substrate with all the components self-aligned in-plane with the lithography resolution, while still maintaining the advantage of combing high speed of operation with long travel range.

## Figures and Tables

**Table 1:** Brief survey of the in-plane electrostatic actuators operated under atmospheric conditions.

Peak Travel Range (µm)	Resonance Frequency	Figure of Merit (µm·kHz)	Peak Translation Velocity (m/s)	References
9.06	26.055 kHz	236.06	1.48	Current work
2	93.5 kHz	187	1.18	
180	769 Hz	138.42	0.87	[11]
115	690 Hz	79.35	0.5	[16]
20	3.8 kHz	76	0.48	[17]
200	329 Hz	65.8	0.41	[2]
112.5	400 Hz	45	0.28	[18]
3	10 kHz	30	0.19	[19]
1	26.48 kHz	26.48	0.17	[20]
37	550 Hz	20.35	0.13	[21]
55	280 Hz	15.4	0.1	[22]
18	465 Hz	8.37	0.05	[23]
14	577 Hz	8.08	0.05	[24]
183.5	30 Hz	5.50	0.03	[25]
18	164 Hz	2.95	0.02	[26]

**Table 2:** Physical dimensions of design A microelectromechanical system (MEMS) actuator.

Physical Dimension	Value
Device thickness	80 µm
Young’s modules	169 × 10^3^ µN/µm^2^
Number of moving fingers	56
Spring beams width	8 µm
Spring beams length	360 µm
Spring truss width	13 µm
Finger length	40 µm
Finger width	3 µm
Air gap between moving and fixed finger	5 µm
Anchors	150 µm × 150 µm

**Table 3:** Physical dimensions of the design B MEMS actuator.

Physical Dimension	Value
Device thickness	80 µm
Number of moving fingers	36
Beams width	10 µm
Beams length	270 µm
Truss width	13 µm
Finger length	30 µm
Finger width	3 µm
Air gap between moving and fixed finger	5 µm
Anchors	150 µm × 150 µm

## Abbreviations

The following abbreviations are used in this manuscript:

MEMS	Microelectromechanical system
SOI	Silicon-on-insulator
DRIE	Deep reactive ion etching
BOX	Buried oxide
FEA	Finite element analysis
FTR	Full travel range
SEM	Scanning electron microscope
FP	Fabry–Perot
SMF	Single-mode fiber
